# A commentary on ‘Genetically proxied intestinal microbiota and risk of bladder cancer’

**DOI:** 10.1097/JS9.0000000000001780

**Published:** 2024-06-10

**Authors:** Xingcheng Zhu, Jianguo He, Junhao Chen

**Affiliations:** a Department of Clinical Laboratory, The Second People’s Hospital of Qujing City Qujing; b Department of Urology, The Second Affiliated Hospital of Kunming Medical University, Kunming, Yunnan Province; c The First Affiliated Hospital of Chongqing Medical University. Yubei District, Chongqing, People’s Republic of China

Highlights

- Mendelian randomization (MR) two-sample analysis should consider non-bidirectional causal relationships. In addition to forward results, reverse MR analysis should also be conducted.
- Large-scale MR analyses should use false discovery rate (FDR) correction to reduce the risk of false positives.
- Numerous studies have conducted mediation analyses between gut microbiota and blood metabolites or immune cells. Further exploration of these mechanisms is warranted, potentially combining multi-omics for transcriptomic analysis.

*Dear Editor,*

We carefully read the article by Zhang *et al*.^1^ published in the *International Journal of Surgery*, which employs two-sample Mendelian randomization (MR) to assess whether there is a causal relationship between gut microbiota and bladder cancer. The main results indicate that certain gut microbiota are risk factors and protective factors for bladder cancer. We commend the authors for their outstanding work and offer some suggestions regarding the experimental design.

Firstly, two-sample MR analysis should consider non-bidirectional causal relationships. It is crucial to conduct reverse MR analysis in addition to assessing forward causal effects. This approach will provide a more comprehensive understanding of causal pathways and ensure the robustness of the findings.

Secondly, for large-scale MR analyses, we recommend using false discovery rate (FDR) correction to mitigate the risk of false positives. FDR correction is essential in large-scale MR analyses to control for the occurrence of false positives and enhance the credibility of the results.

Lastly, numerous studies have involved mediation analysis between gut microbiota and blood metabolites or immune cells. Further exploration of these mechanisms can provide deeper insights into the causal pathways. Additionally, the single nucleotide polymorphisms of these gut microbiota can be converted to genes using tools like the g (https://biit.cs.ut.ee/gprofiler) website. By using Venn diagrams to identify common genes or those with high frequency, followed by transcriptomic analyses such as KEGG (Kyoto Encyclopedia of Genes and Genomes) pathway analysis, potential pathways and mechanisms can be revealed.

We believe that these suggestions can further enhance the clarity and depth of the analysis of gut microbiota and bladder cancer. Once again, we thank Zhang *et al*. for their valuable contributions to this important field.

## Ethical approval

None.

## Consent

None.

## Source of funding

None.

## Author contribution

X.Z.: literature search and writing; J.C.: design; J.H.: provided language polish.

## Conflicts of interest disclosure

The authors declare that they have no conflicts of interest.

## Research registration unique identifying number (UIN)

None.

## Guarantor

Junhao Chen.

## Data availability statement

All data generated and analyzed during this study are included in this published article. The data presented in the article may be requested by consulting the correspondence author.

## Provenance and peer review

None.
